# Effect of Viral Strain and Host Age on Clinical Disease and Viral Replication in Immunocompetent Mouse Models of Chikungunya Encephalomyelitis

**DOI:** 10.3390/v15051057

**Published:** 2023-04-26

**Authors:** Elizabeth J. Anderson, Audrey C. Knight, Mark T. Heise, Victoria K. Baxter

**Affiliations:** 1 Division of Comparative Medicine, University of North Carolina at Chapel Hill, Chapel Hill, NC 27599, USA; 2 Department of Pathology and Laboratory Medicine, University of North Carolina at Chapel Hill, Chapel Hill, NC 27599, USA; 3 Department of Genetics, University of North Carolina at Chapel Hill, Chapel Hill, NC 27599, USA; 4 Department of Microbiology and Immunology, University of North Carolina at Chapel Hill, Chapel Hill, NC 27599, USA; 5 Texas Biomedical Research Institute, San Antonio, TX 78227, USA

**Keywords:** chikungunya virus, encephalomyelitis, alphavirus, mouse model

## Abstract

The alphavirus chikungunya virus (CHIKV) represents a reemerging public health threat as mosquito vectors spread and viruses acquire advantageous mutations. Although primarily arthritogenic in nature, CHIKV can produce neurological disease with long-lasting sequelae that are difficult to study in humans. We therefore evaluated immunocompetent mouse strains/stocks for their susceptibility to intracranial infection with three different CHIKV strains, the East/Central/South African (ECSA) lineage strain SL15649 and Asian lineage strains AF15561 and SM2013. In CD-1 mice, neurovirulence was age- and CHIKV strain-specific, with SM2013 inducing less severe disease than SL15649 and AF15561. In 4–6-week-old C57BL/6J mice, SL15649 induced more severe disease and increased viral brain and spinal cord titers compared to Asian lineage strains, further indicating that neurological disease severity is CHIKV-strain-dependent. Proinflammatory cytokine gene expression and CD4+ T cell infiltration in the brain were also increased with SL15649 infection, suggesting that like other encephalitic alphaviruses and with CHIKV-induced arthritis, the immune response contributes to CHIKV-induced neurological disease. Finally, this study helps overcome a current barrier in the alphavirus field by identifying both 4–6-week-old CD-1 and C57BL/6J mice as immunocompetent, neurodevelopmentally appropriate mouse models that can be used to examine CHIKV neuropathogenesis and immunopathogenesis following direct brain infection.

## 1. Introduction

Chikungunya virus (CHIKV), an Old World alphavirus transmitted via *Aedes aegypti* and *Ae. albopictus* mosquitoes, can be found worldwide and is particularly prevalent in India and Brazil [1]. Classic symptoms of chikungunya (CHIK) include fever, arthralgia, lethargy, headache, and maculopapular rash [2]. However, up to 16.3% of patients also exhibit neurologic symptoms, which can persist for weeks to months after initial infection [3]. These can include encephalitis, myalgia, arthralgia, Horner syndrome, and Guillain–Barre syndrome [4,5,6,7,8,9,10,11]. The neurologic outcomes for children < 1 year old is particularly severe, with around 50% of infants with CHIKV developing symptoms and neurologic delays [12]. Although there has been an increasing frequency of neurologic complications associated with CHIKV infection, studying virus-induced disease in the central nervous system (CNS) is challenging in human patients due to the inaccessibility and impaired regenerative ability of CNS tissue. Therefore, alternative approaches, such as animal models, are required to study CHIKV neuropathogenesis.

Mice represent a valuable animal model for studying alphavirus pathogenesis due to their small size, the availability of a large number of mouse-specific reagents, and their relative ease of genetic manipulation. Much of the knowledge regarding alphavirus encephalomyelitis pathogenesis has been gained by studying Sindbis virus (SINV), the prototypic alphavirus, in mice [13,14,15]. In contrast, mouse models used for CHIKV infection have focused largely on the arthritis disease phenotype produced when CHIKV is subcutaneously (SQ) injected via the footpad in mice. While a few papers have detected CHIKV in the brains of adult C57BL6/J (B6) mice following SQ or intranasal (IN) infection [16,17,18], most previous studies evaluating neurologic disease have used neonatal mice or immune-compromised strains, such as type-I interferon (IFN) knockouts [19,20,21,22,23,24,25,26,27,28,29,30,31,32,33,34]. However, development and characterization of CHIKV infection in immunocompetent mouse models that are neurodevelopmentally equivalent to susceptible human populations are critical for studying disease mechanisms in the CNS.

There are three phylogenetic lineages of CHIKV: West African, Asian, and East/Central/South African (ECSA). The West African strain is more geographically isolated than other CHIKV lineages, primarily circulating in Senegal, and tends to result in smaller outbreaks [35,36,37]. However, high levels of viremia and clinical disease have been reported following infection with West African strains in mice and macaques [38,39]. Asian and ECSA lineages have a wider geographic range and are responsible for most of the CHIKV outbreaks worldwide [35,40,41,42,43]. CHIKV clades vary not only in geographic distribution, but also in disease severity based on both human clinical case studies [41,42,43] and experimental mouse studies focusing on footpad swelling phenotypes [38,44]. ECSA lineage CHIKV strains have been reported to cause more severe disease and long-term persistence in humans [45]. However, CHIKV genotyping is rarely performed in most human cases, so epidemiological tracing may be inconsistent, and neurological disease has been reported following infection with Asian lineage CHIKVs in both mice and humans [12,23]. Additionally, CHIKV frequently circulates in areas also endemic to dengue, yellow fever, and Zika viruses, which can have similar clinical phenotypes and may not be easily distinguished from CHIKV infection.

In this study, we evaluated three strains of CHIKV, one ECSA lineage strain and two Asian lineage strains, in an outbred mouse stock and inbred mouse strain to establish the relative severity of neurological disease across multiple stages of neurodevelopment in immunocompetent mice. CHIKV-induced neurological disease was found to be CHIKV- strain-dependent, with infection with the ECSA lineage strain resulting in more severe neurological disease, higher viral titers in the brain and spinal cord, and increased proinflammatory gene expression and CD4+ T cell infiltration in the brain in 4–6-week-old mice. Furthermore, through this work, we determined that both 4–6-week-old CD-1 and B6 mice may serve as immunocompetent, neurodevelopmentally equivalent mouse models for the study of CHIKV infection in the CNS.

## 2. Materials and Methods

### 2.1. Ethics Statement

All procedures were conducted under an animal use protocol approved by the Institutional Animal Care and Use Committee at the University of North Carolina at Chapel Hill (protocol numbers 17-024 and 19-288). All animal work was performed in strict accordance with recommendations outlined in the Guide for the Care and Use of Laboratory Animals and in compliance with the concept of the “3 R’s”. All live CHIKV work was performed under BSL3 conditions at the University of North Carolina at Chapel Hill in strict adherence to safety precautions outlined in Biosafety in Microbiological and Biomedical Laboratories (BMBL).

### 2.2. Virus Stocks

CHIKV stocks were made from infectious clones from clinical isolates AF15561 (GenBank: EF452493.1) provided by Thomas Morrison’s laboratory at the University of Colorado Anschutz School of Medicine, and SM2013 (GenBank: MT228631.1) and SL15649 (GenBank: GU189061.1) provided by the Heise laboratory at the University of North Carolina at Chapel Hill. An infectious clone of SINV TE was provided by Diane Griffin’s laboratory at the John Hopkins Bloomberg School of Public Health. Virus stocks were grown in culture by electroporating RNA into BHK-21 cells (ATCC stock CCL-10) and clarifying the supernatant collected at 36 h post-electroporation. Stock titers were established by plaque assay on Vero-81 cells (ATCC stock CCL-81).

### 2.3. Mouse Infections

During experiments, mice were housed in an ABSL3 facility on a 12:12 light cycle. Autoclaved cages (Tecniplast, 2000P or EM500) were used with irradiated Bed-o-Cob (ScottPharma, Bed-o-Cob 4RB), ad libitum irradiated chow (LabDiet, PicoLab Select Rodent 50 IF/6F 5V5R) and autoclaved water bottles. Cages were changed every 14 days, and water bottles were changed every 7 days. For CD-1 mouse studies, timed pregnant female mice were purchased from Charles River (Stock 022) and allowed to give birth in the ABSL3 facility, with the resulting pups used in infection experiments. For postnatal (PN) infections at 2, 7, and 14 days of age, litters were randomized across infection groups, with litter sizes ranging from 9–15 pups (mean = 12 ± 2 pups). For mice infected at PN28, mice were weaned at PN21 and randomized into cages for infection. For C57BL/6J (B6) mouse studies, male and female breeders were ordered from Jackson Labs (Stock 000664) and bred in-house. B6 mice were infected at 4–6 weeks of age. Male and female mice were used in all experiments, with both sexes represented in each group at each collection timepoint. Separate 4–6-week-old B6 mice were ordered for use as PBS mock IC-infected controls for qPCR studies.

Infections were all performed under light isoflurane (Baxter, Deerfield, IL, USA) anesthesia. Intracranial (IC) infections were performed with 1000 plaque forming units (PFU) diluted in 10 µL (PN2, PN7, PN14 CD-1 mice) or 20 µL (PN28 CD-1 and B6 mice) sterile PBS using a 29G insulin U-100 syringe introduced into the left hemisphere of the brain. Footpad infections were performed by SQ injecting 1000 PFU in 10 µL PBS into the left hind footpad using a 29G insulin U-100 syringe. IN infections were performed by pipetting 10^5^ PFU in 20 µL sterile PBS across both nares. Plaque assays were performed on diluted virus solutions immediately following each infection to confirm infection doses.

Neonatal CD-1 mice (PN2, PN7, and PN28 infection groups) were visually checked twice a day for morbidity and mortality and weighed at 0, 7, and 14 days post infection (DPI). Once a day, PN28 CD-1 and B6 mice were weighed and clinically evaluated using a clinical scoring system adapted from a scoring system developed for SINV TE [46]: score of 0 = no clinical signs; 1 = mild abnormal gait and tail posture; 2 = hunched posture, abnormal gait, unkempt hair coat; 3 = marked hunched posture, ataxia, and decreased ambulation (humane endpoint); 4 = moribund/dead/euthanized. Investigators were blinded to the infection status or viral strain group during clinical scoring.

### 2.4. Tissue Collection

Animals were euthanized using isoflurane overdose and perfused with 10–15 mL ice-cold PBS. Brains and spinal cords were collected, flash frozen in liquid nitrogen, and stored at −80 °C. For immunohistochemical (IHC) studies, mice were perfused with 40 mL 4% paraformaldehyde (PFA) and brains were removed and immersed in 4% PFA at 4 °C. After 24 h, brains were rinsed in 1× PBS and stored in 1× PBS until processing.

### 2.5. Quantification of Infectious Virus

Brains and spinal cords were homogenized in ice-cold PBS to 20% *w*/*v* and 10% *w*/*v* concentrations, respectively. Homogenates were clarified at 10,000 RPM for 15 min then diluted from 10^−1^ to 10^−6^ (brain) or 0.5 to 10^−5^ (spinal cords) for plaque assays. An amount of 200 µL of homogenates dilutions were plated in duplicate over a monolayer of Vero-81 cells (ATCC CCL-81) in 12-well cell culture plates. Plates were incubated at 37 °C, 5% CO_2_ with periodic gentle rocking for 1 h before being overlaid with 1× αMEM (Corning) containing 0.2 mM L-glutamine (Gibco), 5% FBS (Atlanta Biologicals), 1 mM HEPES (Corning), 1% Penicillin-Streptomycin (Gibco), and 1.25% carboxymethylcellulose sodium (Sigma). Plates were then incubated for 48 h at 37 °C, 5% CO_2_ before being fixed with 4% PFA, washed with water, and stained with 0.25% crystal violet (VWR). The limit of detection (LOD) for the assay was determined to be 125 (2.1 log10) PFU/g tissue for brain and 50 (1.7 log10) PFU/g tissue for spinal cord, and samples that fell below the LOD were assigned a titer of one half of the LOD. When reporting inoculation virus amounts on the brain viral titer graphs to assure active virus replication, average brain weights of each mouse age were taken into account. The average brain weight of a PN2 CD-1 mouse was 200 mg, so inoculation dose was presented as 5000 (3.7 log10) PFU/g tissue. The average brain weight of a PN28 CD-1 mouse was 580 mg, so inoculation dose was presented as 1725 (3.2 log10) PFU/g tissue. The average brain weight of a 4–6-week-old B6 mouse was 500 mg, so inoculation dose was presented as 2000 (3.3 log10) PFU/g tissue.

### 2.6. RNA Isolation and Quantitative PCR

Flash-frozen brains collected at necropsy were placed in tubes with glass beads and 1 mL of TRIzol (cat. 15596026, Invitrogen, Waltham, MA, USA) and homogenized. Samples were then centrifuged at 15,000× *g* for 15 min at 4 °C, and then the clarified supernatant was transferred to another tube. RNA was isolated from said samples using the RNeasy Lipid Tissue Mini Kit (cat. 74804, Qiagen, Germantown, MD, USA) with a chloroform separation according to the manufacturer’s protocol. A 15 min DNase I (cat. 79254, Qiagen, Germantown, MD, USA) digest was used to purify samples. cDNA was generated using 1 µg of RNA and the high-capacity cDNA reverse transcription kit according to the manufacturer’s protocol (cat. 4368814, Applied Biosystems, Waltham, MA, USA). For the qPCR reaction, the TaqMan Fast Advanced Master Mix (cat. 4444963, ThermoFisher, Waltham, MA, USA) was used according to the manufacturer’s protocol. An amount of 2 µL of cDNA and 1 µL of primer probe were added to each reaction. The reaction was run using the QuantStudio 3 thermocycler (cat. A28566, Applied Biosystems, Waltham, MA, USA). A limit of detection of 34 Ct was set for all primer probe sets. For analysis, all Ct values were normalized to *Gapdh*. Values are reported as fold change (2^−ΔΔCt^) compared to mock infected samples. A list of primer probes used can be found in Supplemental Table S1.

### 2.7. Histopathology

PFA-fixed brains were sectioned coronally (Zivic Instruments, Pittsburgh, PA, USA) and then embedded in paraffin blocks. Sections of 5 µm were then taken for immunohistochemistry on the Leica Bond III Autostainer system on positively charged slides (Leica, LOC, Wetzlar, Germany). Dual Chromogenic Immunohistochemistry was accomplished using CD4 (ab183685, clone EPR19514, abcam, Boston, MA, USA) and CD8 (14-0808-82, clone 4SM15, Invitrogen, Waltham, MA, USA) antibodies on the Ventana Discovery platform (Roche, Indianapolis, IN, USA). Slides were dewaxed and hydrated. Heat-induced antigen retrieval was accomplished using Ventana’s CC1 (pH 8.5). After pretreatment, slides were incubated with primary antibodies as follows: CD8 at 1:100 and CD4 1:1000, using Discovery Casein Diluent (760-219, Roche, Indianapolis, IN, USA). Ready-to-use secondary antibodies Discovery OmniMap anti-Rabbit HRP (760-4311, Roche) or Discovery OmniMap anti-Rat HRP (760-4457, Roche, Indianapolis, IN, USA) were used, followed by DAB or Discovery Purple (760-229, Roche, Indianapolis, IN, USA) development and Hematoxylin II staining. Stained slides were dehydrated and coverslipped with Cytoseal 60 (8310-4, Thermo Fisher Scientific, Waltham, MA, USA). A positive control slide containing mouse spleen tissue with known CD4+ and CD8+ T cells was included each time a set of slides was stained using the Autostainer.

Nikon Elements Advanced Research software (version 4.60) was used to perform quantitative image analysis. Images were captured at 200× and tiled together in order to fit approximately the same area of the corpus callosum into the area analyzed. A region of interest (ROI) was set around the corpus callosum, excluding other anatomic regions. A threshold was determined for both DAB (brown) and purple (Ventana). An object count was then determined based on area, circularity, and equal diameter of the cell. From this, data were reported as CD4+/CD8+ per µm.

### 2.8. Statistics

Statistics were performed using GraphPad Prism 9 software. Survival data were analyzed by Mantel Cox test. Weight loss data were analyzed by two-way ANOVA with Dunnett multiple comparisons test. Titer and CNS inflammation data were analyzed by two-way ANOVA with Sidak’s or Tukey’s multiple comparisons tests. A *p* value of <0.05 was considered significant for all analyses.

## 3. Results

### 3.1. Outbred CD-1 Mice Demonstrate Age- and Viral Strain-Dependent Clinical Outcomes Following Intracranial CHIKV Infection

Previous work examining neurological infection in mice with other alphaviruses SINV and Semliki Forest virus (SFV) has shown that susceptibility to disease development is age-dependent, with younger animals, especially neonates, demonstrating higher mortality [13,47,48]. We sought to determine whether neurological infection with CHIKV resulted in similar age-dependent susceptibility to clinical disease and mortality and whether that susceptibility was CHIKV strain-dependent. Three CHIKV strains were examined: one ECSA-lineage strain: SL15649, isolated from human serum in Sri Lanka in 2006 [49], and two Asian-lineage strains: AF15561, the pathogenic parental strain to the attenuated 181/25 CHIKV strain isolated from an infected patient in Thailand [34], and SM2013, isolated in 2013 from St. Martin in the Caribbean [50].

**Figure 1:**
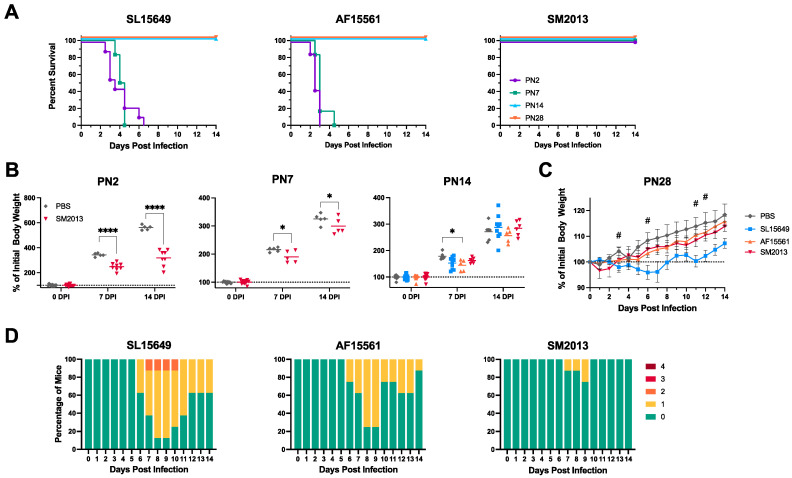
Clinical disease in CD-1 mice infected IC with different CHIKV strains at various ages. (**A**) Mortality was measured daily in CD-1 pups infected at 2, 7, 14, and 28 days postnatal (PN) with CHIKV strains SL15649 (**left**), AF15661 (**center**), and SM2013 (**right**); *n* = 12−15 mice per infection group per age at infection. (**B**) Body weights were measured at 0, 7, and 14 DPI in mice infected at PN2 (**left**), PN7 (**center**), and PN14 (**right**), shown as percent of group’s average 0 DPI weight; dotted line represents initial starting weight; solid horizontal line represents data mean; * *p* < 0.05, **** *p* < 0.0001, Sidak’s (PN2, PN7) or Dunnett’s (PN14) multiple comparisons test. (**C**) Body weights were measured daily in mice infected at PN28, shown as percent of each mouse’s initial body weight at infection; *n* = 8 mice per group; dotted line represents initial starting weight; data presented as mean ±SEM; # *p* < 0.05 for PBS vs. SL15649, Dunnett’s multiple comparisons test. (**D**) Clinical scores were recorded daily for mice infected at PN28 with SL15649 (**left**), AF15561 (**center**), and SM2013 (**right**); *n* = 8 mice per group; 0 = no clinical signs, 1 = mild abnormal gait and tail posture, 2 = hunched posture, abnormal gait, unkempt hair coat, 3 = marked hunched posture, ataxia, and decreased ambulation (humane endpoint), 4 = moribund/dead/euthanized.

CD-1 mice, an outbred immunocompetent mouse stock known for producing large litter sizes and providing good maternal care [51,52], were inoculated with 1000 PFU of one of each of the CHIKV strains across a range of postnatal (PN) days and observed for clinical disease development (Figure 1). Significant differences in mortality across ages were observed in mice infected with SL15649 and AF15561 (*p* < 0.0001, Mantel-Cox), but not SM2013 (Figure 1A). For pups infected with SL15649 and AF15561 at PN2 and PN7, 100% mortality was observed by 6.5 days post infection (DPI). In contrast, no mortality was observed at any age in mice infected with SM2013, or in mice infected at PN14 and PN28 with any CHIKV strain. However, pups infected with SM2013 at PN2 and PN7 showed significantly impaired weight gain compared to mock-infected controls (Figure 1B), indicating SM2013 infection was not innocuous. In contrast, with infection at PN14, only AF15661-infected pups at 7 DPI showed mildly reduced weight gain compared to mock-infected controls; by 14 DPI, all PN14 pups showed comparable weight gain.

When infected post-weaning at 4 weeks of age (PN28), mice were monitored daily for weight change and neurological disease, adapting a scoring system previously developed for mice infected with the nonfatal TE strain of SINV [46]. Of the CHIKV strains, only SL15649 induced weight loss and significant weight change relative to PBS mock-infected controls from 6 to 14 DPI (*p* = 0.0284) (Figure 1C). Duration and severity of neurological scores varied between CHIKV strains (Figure 1D). SM2013 induced minimal clinical disease, with only two animals scoring above a ‘0’ from 7 to 9 DPI. SL15649 and AF15561 induced scores of at least ‘1’ in most mice, with some SL15649-infected mice reaching a score of ‘2’ from 7 to 10 DPI. These data show that outbred, immunocompetent mice are readily infected by multiple strains of CHIKV, with resulting disease dependent on age and virus strain.

### 3.2. Viral Replication in the Brain Differs Based on Age of Infection and CHIKV Strain in CD-1 Mice

**Figure 2:**
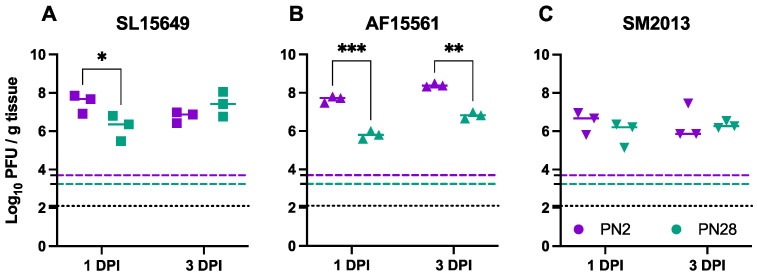
Brain CHIKV titers in CD-1 mice infected IC with different CHIKV strains at different ages. Infectious virus levels in the brain of CD-1 mice intracranially infected with CHIKV strains SL15649 (**A**), AF15561 (**B**), and SM2013 (**C**) at PN2 or PN28; dotted black lines represent assay limit of detection; dashed purple line and dashed green line represent input virus amounts for PN2 and PN28 mice, respectively, normalized to the average brain weight at each age; solid horizontal purple and green lines represent data median; * *p* < 0.05, ** *p* < 0.01, *** *p* < 0.001, Sidak’s multiple comparisons test.

Like with clinical disease, age has been shown to indirectly correlate with virus replication in the brains of mice with other alphaviruses [13,24,47,53,54]. To see if this extended to CHIKV, viral titers were measured in the brains of CD-1 mice infected with each of the three CHIKV strains at PN2 and PN28. Virus replication in the brain was robust, with all three viruses replicating to over 100× higher titers than the infection dose at 1 DPI (Figure 2). Brain titers at 1 DPI in mice infected with SL15649 at PN2 were significantly higher than those infected at PN28 but comparable at 3 DPI (Figure 2A). AF15561 infection resulted in significantly higher titers in PN2-infected mice versus PN28-infected mice at both 1 and 3 DPI (Figure 2B). In contrast, age did not affect SM2013 titers at either 1 or 3 DPI (Figure 2C). Therefore, age-dependent restriction of virus replication is CHIKV strain-dependent in mice.

### 3.3. Four-To-Six-Week-Old C57BL/6J Mice Are Susceptible to CHIKV-Induced Clinical and Neurological Disease

Since outbred CD-1 mice were susceptible to CHIKV infection but showed a variable clinical response depending on the CHIKV strain used, we next wanted to determine how a young adult, immunocompetent inbred mouse strain would respond to CHIKV infection. C57BL/6J (B6) mice represent an attractive animal model for studying CHIKV neuropathogenesis, as the strain is commonly used to study CNS viral infections and alphavirus pathogenesis [46,49,55,56,57,58,59,60], is the reference mouse genome, is the background strain of most genetically modified mice, and has an extensive genetic and immunological tool set by which to evaluate mechanisms of disease development. Four-to-six-week-old C57BL/6J mice were IC infected with the same 1000 PFU dose of CHIKV SL15649, AF15561, or SM2013. CHIKV-induced clinical disease was compared to mice mock-infected with PBS or infected with 1000 PFU SINV TE, a nonfatal strain of the prototypic alphavirus with well-characterized neurological disease progression in B6 mice.

**Figure 3:**
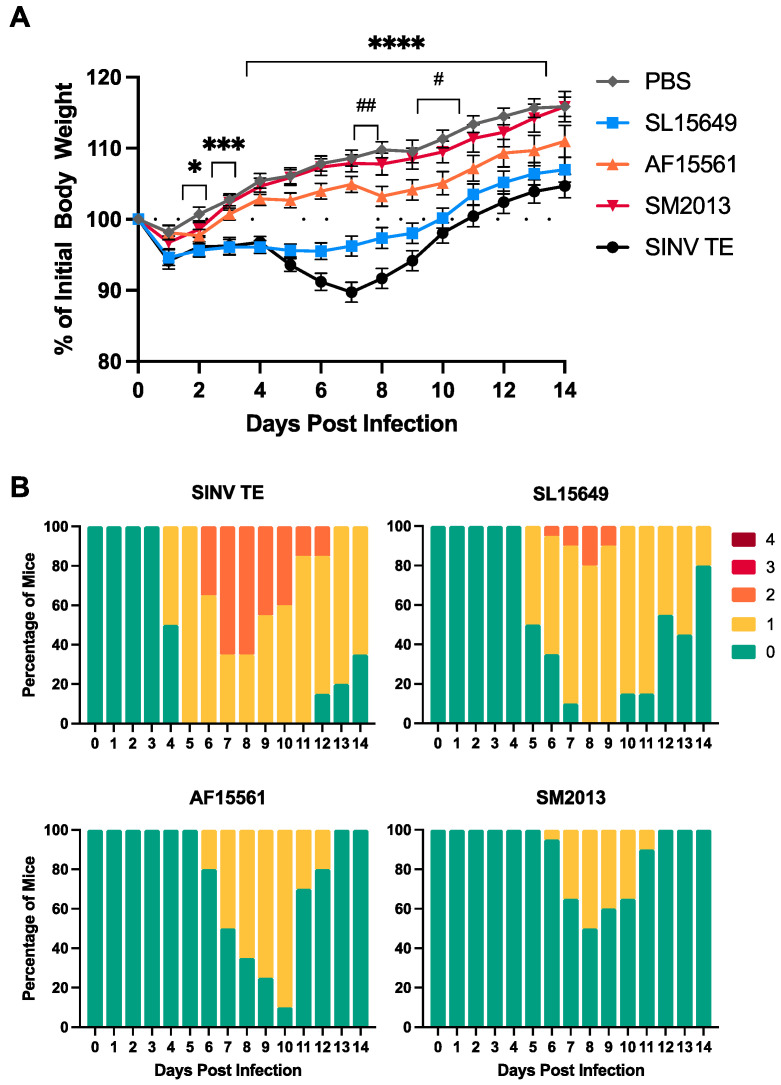
Clinical disease in C57BL/6 mice infected IC with different CHIKV strains. Four-to-six week old C57BL/6J mice were IC infected with 1000 PFU of SINV TE or CHIKV strains SL15649, AF15561, or SM2013 or mock infected with PBS and weighed (**A**) and clinically scored (**B**) daily; for (**A**), *n* = 46 mice per group for SL15649, AF15561, and SM2013, *n* = 31 mice per group for PBS Mock and SINV TE; data presented as mean ± SEM; dotted line represents initial starting weight; Mock versus SL15649: * *p* < 0.05, *** *p* < 0.001, **** *p* < 0.0001; Mock versus AF15561: # *p* < 0.05, ## *p* < 0.01, Dunnett’s multiple comparisons test; for (**B**), *n* = 20 mice per group; 0 = no clinical signs, 1 = mild abnormal gait and tail posture, 2 = hunched posture, abnormal gait, unkempt hair coat, 3 = marked hunched posture, ataxia, and decreased ambulation (humane endpoint), 4 = moribund/dead/euthanized.

Unlike suckling CD-1 pups, but similar to PN14- and PN28-infected CD-1 mice, CHIKV did not induce any mortality in B6 mice. Weight was not significantly affected by AF15561 or SM2013 infection (Figure 3A). In contrast, SL15649 infection resulted in significant weight loss relative to PBS mock-infected controls, demonstrating approximately 5% weight loss through 10 DPI. However, infection with CHIKV resulted in less weight loss compared to SINV TE.

Clinical scores followed a similar pattern to that seen in PN28-infected CD-1 mice (Figure 3B). Mice infected with SL15649 demonstrated clinical scores above ‘0’ from 5 to 14 DPI, with some transiently reaching a clinical score of ‘2’. However, scores did not reach the same magnitude as SINV TE-infected mice, where 50% reached a clinical score of ‘2’ by 7 DPI, and more than half were still scoring a ‘1’ by the end of the study at 14 DPI. AF15561- and SM2013-infected mice showed less severe clinical sign development, with no mice progressing past a clinical score of ‘1’. Similar to young-adult CD-1 mice, in B6 mice, the ECSA-lineage strain SL15649 induced more severe disease than Asian lineage strains AF15561 and SM2013.

### 3.4. Viral Titers in Brain and Spinal Cord Vary by CHIKV Strain and Inoculation Route in C57BL/6 Mice

**Figure 4:**
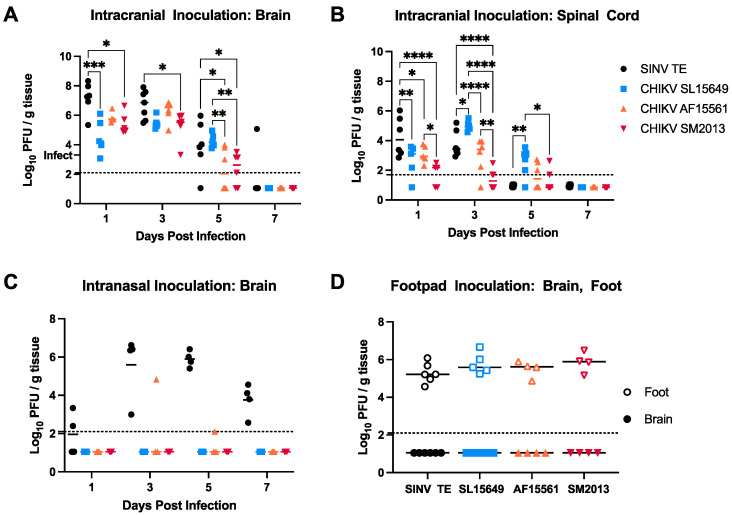
CNS Titers in C57BL/6 mice infected with different CHIKV strains via different infection routes. (**A**–**C**) Mice were infected IC with 1000 PFU (**A**,**B**) or IN with 105 PFU (**C**) of SINV TE or CHIKV SL15649, AF15561, or SM2013, and titers were performed by plaque assay in brains (**A**,**C**) and spinal cords (**B**) collected at 1, 3, 5, or 7 DPI. (**D**) Mice were infected with 1000 PFU of SINV TE or CHIKV SL15649, AF15561, or SM2013 by subcutaneous footpad, and titers were performed by plaque assay in ipsilateral feet (closed symbols) and brains (open symbols) collected at 5 DPI; dotted horizontal lines represent assay limit of detection; for (**A**), “Infect” tick mark on *y*-axis represents input virus amount; * *p* < 0.05, ** *p* < 0.01, *** *p* < 0.001, **** *p* < 0.0001, Tukey’s multiple comparisons.

Clinical disease development indicated that B6 mice are susceptible to CHIKV infection following direct IC inoculation, and to evaluate the amount of virus present, viral titers were performed by plaque assay through 7 DPI. While brain titers exceeded the amount of input virus and peaked at 1 to 3 DPI, SL15649-infected mice took longer to clear infectious virus from the brain, with titers remaining significantly higher at 5 DPI compared to AF15561- and SM2013-infected mice, similar to SINV-infected brains (Figure 4A). Spinal cord titers were overall approximately 10× lower compared to brains, and virus was only sporadically detected in spinal cords of SM2013-infected mice (Figure 4B). While titers in SINV-infected spinal cords were significantly higher than the three CHIKV strains at 1 DPI, at 3 and 5 DPI, SL15649-infected spinal cords showed significantly higher titers than even SINV, suggesting enhanced myelotropism.

Our findings thus far have indicated that 4–6-week-old B6 mice are susceptible to CNS infection with multiple CHIKV strains following IC (i.e., direct brain) inoculation; however, IC inoculation does not replicate the natural course of CHIKV infection seen in humans or allow investigators to study the mechanisms and dynamics behind CHIKV neuroinvasion. We therefore next performed indirect infections by IN and SQ footpad inoculations to see if CHIKV could successfully infect the CNS from the periphery in B6 mice. Aside from SINV, which is known to invade the CNS by axonal transport up the olfactory bulb in mice [61,62], all three CHIKV strains failed to consistently produce titers in brain tissue following IN inoculation (Figure 4C). Similarly, while virus was readily detectable at 5 DPI in ipsilateral feet following SQ footpad injection, no virus was detected in the brain (Figure 4D). This suggests that while these three clinical CHIKV strains can readily infect the brain and, in most cases, spinal cord following direct IC infection, CNS infection from the periphery is restricted in 4–6-week-old B6 mice.

### 3.5. The Immune Response in the Brain to IC CHIKV Infection Is CHIKV Strain Dependent

**Figure 5:**
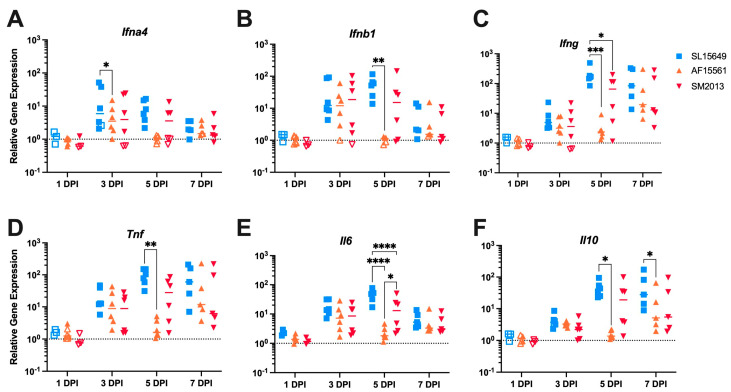
Expression of interferons and inflammatory cytokines in mouse brains infected IC with CHIKV. Relative expression of *Ifna4* (**A**), *Ifnb1* (**B**), *Ifng* (**C**), *Tnf* (**D**), *Il6* (**E**), and *Il10* (**F**) in the brains of 4–6-week-old B6 mice following IC CHIKV infection was measured by qRT-PCR. Open symbols represent samples with one or both replicates below the assay limit of detection; dotted horizontal lines represent gene expression of mock-infected control brains; * *p* < 0.05; ** *p* < 0.01; *** *p* < 0.001; **** *p* < 0.0001, Tukey’s multiple comparisons.

Neurological disease development and CNS damage following SINV and SFV infection and arthritis following CHIKV infection are known to be primarily mediated by the immune response rather than directly by viral replication [63,64,65,66,67]. We therefore sought to determine if the different CHIKV strains showed different IFN and cytokine expression profiles in the brain throughout the course of CHIKV infection in IC-infected B6 mice (Figure 5). Genes for IFN-α (Figure 5A) and IFN-β (Figure 5B) were significantly upregulated in SL15649-infected brains compared to AF15561-infected brains at 3 and 5 DPI, respectively. *Ifng* expression was also significantly upregulated in SL15649-infected brains compared to both AF15561 and SM2013 at 5 DPI (Figure 5C). Similarly, SL15649-infected brains showed significant upregulation of proinflammatory cytokine genes *Tnf* (Figure 5D) and *Il6* (Figure 5E) at 5 DPI; however, interestingly, *Il6* expression was also upregulated in SM2013-infected brains compared to AF15561-infected brains at 5 DPI. Finally, gene expression of the regulatory cytokine IL-10 was also upregulated in SL15649-infected brains compared to AF15561-infected brains at both 5 and 7 DPI (Figure 5F). These results indicate that SL15649 induces a more robust proinflammatory cytokine response in the brain compared to the Asian lineage strains, correlating with more severe disease development.

**Figure 6:**
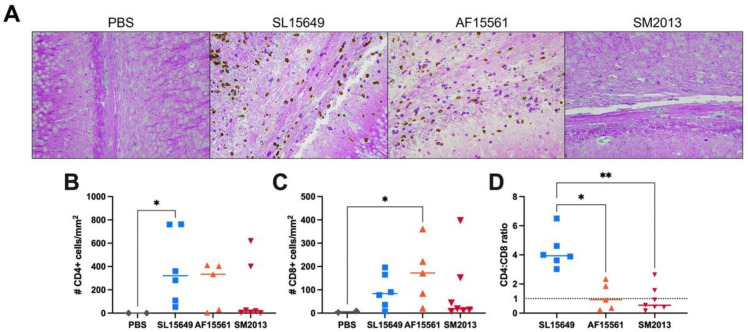
CD4+ and CD8+ T cell infiltration to the corpus callosum of B6 mice following CHIKV infection. Four-to-six-week-old B6 mice were infected IC with CHIKV SL15649, AF15561, or SM2013 or mock infected with PBS, and brains were collected at 7 DPI for co-staining for CD4+ and CD8+ T cells by IHC. (**A**) Representative 200× photomicrographs of the corpus callosum area of B6 mouse brains showing CD4+ (brown) and CD8+ (bright purple) T cells. CD4+ T cells (**B**) and CD8+ T cells (**C**) were quantified in the corpus callosum, and the ratio of CD4:CD8 was calculated for each mouse (**D**). For (**D**), dotted horizontal line represents an equal ratio of CD4+ to CD8+ T cells; * *p* < 0.05; ** *p* < 0.01; Dunn’s multiple comparisons tests.

T lymphocytes are known to play an important role in disease pathogenesis in the SINV mouse model of alphavirus encephalomyelitis, with infiltration peaking in the brain at 7 DPI following IC infection [67,68]. To evaluate the effect of CHIKV infection on T cell infiltration into the brain, dual CD4/CD8 IHC was performed on PFA-fixed, paraffin-embedded brain sections at 7 DPI. Occasional CD8+ T cells, but no CD4+ T cells, were detected in the corpus callosum of mock-infected mice. In contrast, while most SM2013-infected mice also had minimal T cell infiltration, CD8+ and CD4+ T cells were readily detectable in both AF15561 and SL15649-infected brains (Figure 6A). SL15649 infection resulted in a significant increase in CD4+ T cells within the corpus callosum (Figure 6B), while AF15561 infection caused a significant increase in CD8+ T cells compared to mock PBS-infected controls (Figure 6C). Interestingly, the ratio of CD4+:CD8+ T cells was significantly higher in mice infected with SL15649, whereas both SM2013 and AF15561 had ratios closer to 1 (Figure 6D); this indicates SL15649 induces a stronger CD4+ T cell response to neurological CHIKV infection than the Asian lineage strains.

## 4. Discussion

Our studies found that both 4–6-week-old CD-1 and B6 mice are susceptible to IC infection with multiple clinical strains of CHIKV, presenting animal models that reflect the immune and neurodevelopmental status of susceptible human populations by which to study CHIKV neuropathogenesis. Several animal models are available for studying CHIKV-induced arthritogenic disease, but a limited number of options are available for the study of neurologic involvement. The few studies using non-human primates have shown them to have similar features of neurologic involvement compared to humans [69,70]; however, due to their housing and cost constraints, non-human primates are more often used for studies evaluating potential countermeasures rather than to study atypical manifestations or pathogenesis of CHIKV infection. Zebrafish have been used for genetic manipulation and CHIKV dissemination and tissue tropism studies [61,71]; however, the lack of a mammalian immune system presents a limitation to studying the immunopathogenesis of CHIKV infection. The small size, relative inexpensiveness, similarity of the immune system and anatomy to humans, availability of reagents, and relative ease of genetic manipulation make mice an extremely attractive small mammalian model for studying CHIKV neuropathogenesis; however, thus far, most studies have either used immunocompromised [24,25,33] or neonatal mice [19,21,22,23,24,25,26,27,28,29,30,31,32,34] to achieve CNS infection. In particular, using neonatal mice to study CHIKV neuropathogenesis presents translatability issues outside of congenital infections, as PN2 mice are neurodevelopmentally equivalent to a third trimester fetus and PN7 to a near-term fetus or newborn infant [72]. In contrast, 4–6-week-old mice are neurologically equivalent to a child of 1–2 years [72], a highly susceptible age to neurological CHIKV infection [12]. These factors drove us to look at CD-1 and B6 mice as immunocompetent and neurologically relevant models of neurological CHIKV infection, and to our knowledge, this study represents the first comparison of clinical CHIKV strains from multiple phylogenetic clades in their propensity to infect the CNS and cause neurological disease using such models.

CD-1 and B6 mice each possess characteristics that are advantageous when approaching different research questions, and these characteristics guided our decisions in which strain or stock to use for the different experiments presented in this study. CD-1 mice produce large litters and are outstanding mothers, making them ideal models for performing age-related studies involving neonatal mice [51,52]. Additionally, their outbred nature makes them less expensive in general than inbred mouse strains, which require genetic management to ensure genetic drift is minimized, and provides genetic diversity to studies, which is important for translating potential outcomes in humans, especially when evaluating potential vaccines or treatments. Inbred B6 mice provide advantages when performing more mechanistic studies, particularly in studying the immune response to infection, as they are the reference mouse genome and background strain for most gene knockout and genetically modified lines [73]. Furthermore, many pathogenesis studies with other encephalitic alphaviruses have been performed using B6 mice [46,58,59,60], allowing for comparisons across the viral genus, and their inbred nature tends to reduce variability in responses and data. While more work is needed to determine the full relevance of each model to translating human disease, having two mouse stocks or strains in which to study neurological CHIKV infection presents the option to cross-check findings from one model in the other and evaluate disease pathogenesis in a more comprehensive manner.

CHIKV strains from different phylogenetic lineages have been reported to produce varied clinical outcomes in human patients [41,42,43], in line with our findings here. ECSA and Asian-lineage CHIKVs have demonstrated differences in mortality rates in neonatally infected ICR mice, with the Asian-lineage virus inducing more severe pathology associated with an enhanced immune response [23]. Similarly, mice infected neonatally via footpad infection showed differences between strains in arthritogenic phenotypes [38,44]. In humans, many reports of high neurological disease prevalence have come from areas where the ECSA lineages dominantly circulate [3], and previous studies reporting successful CHIKV neuroinvasion in adult B6 mice all used ECSA lineage CHIKVs [16,17,18]. Consistent with this, in our study, while several 4–6-week-old CD1 and B6 mice infected with Asian-lineage strains AF15561 and SM2013 demonstrated clinical signs, they were not as severe as with the ECSA-lineage SL15649 strain. SL15649 also showed the slowest viral clearance and was the most myelotropic of the tested strains in B6 mice. Interesting, a difference in neurovirulence between the two Asian-lineage strains was found, with SM2013 showing minimal neurovirulence and myelotropism and AF15561 replicating to the highest titers in the B6 mouse brains of the CHIKV strains; this suggests that CHIKV neurovirulence differences cannot be attributed to phylogenetic clade classification alone.

Host age and neurodevelopmental status have been shown to play a role in susceptibility to alphavirus infection and disease severity, with CNS infection shown to be lethal in neonatal, but not weanling, mice [13,47,48,74]. In our studies, CD-1 mice infected with CHIKV strains SL15649 and AF15561, but not SM2013, showed similar host age-related mortality, with 100% mortality observed in suckling mice (PN2 and PN7), but 0% mortality in PN14 or PN28 mice. With other alphaviruses, this difference in susceptibility and virulence has been shown to be due to maturation of the CNS rather than that of the immune response [13,48], with neuronal maturation associated with restricted viral RNA and protein production and increased antiviral signaling [53,75,76]. In consonance with this, mice infected with CHIKV SL15649, and especially AF15649, showed an age-dependent decrease in viral titers in the brain from PN2 to PN28. This suggests that CHIKV similarly shows an age-dependent restriction in neurovirulence, and further examination of the viral and host factors that contribute to neurovirulence is warranted.

Alphavirus pathogenesis, both with arthritogenic and neurological disease, has been shown to primarily be mediated by the immune response to infection rather than directly due to viral replication [77]. In the SINV mouse model of alphavirus encephalomyelitis, development of neurological disease and pathology coincides with virus clearance and infiltration of inflammatory cells into the CNS [67,78,79]. Proinflammatory cytokines, including IFN-γ and TNF-α, and T cells, particularly CD4+ T cells, mediate neurological disease development and mortality in SINV-infected mice [63,68,79,80,81,82,83]. While less work has evaluated the role of the immune response in neurological CHIKV infection, evaluation of neonatal mice infected with CHIKV found differential expression of immune response genes that correlate with CNS pathology and mortality [23,30]. We found that expression of interferon and proinflammatory cytokines and numbers of CD4+ T cells were increased in the brains of SL15649-infected mice relative to mice infected with Asian-lineage strains. As IC infection with SL15469 produced more severe disease, as evidenced by weight loss and consistently elevated neurological disease scores, concurrent with elevation of proinflammatory cytokine expression but following peak viral brain titers, this suggests that similar to other neurotropic alphaviruses and arthritogenic CHIKV, disease induced by neurological CHIKV infection is mediated by the immune response. Furthermore, CD8+ T cell responses play a key role in viral clearance, whereas a dominant CD4+ T cell response has been shown to be a major feature of arthritogenic and chronic CHIKV infection [64,84]. This suggests that the skew toward a CD4+ T cell-dominant rather than a CD8+ T cell response may be contributing to the severe disease and delayed clearance of virus from the brains of SL15649-infected mice. Further studies characterizing the T cell response more in depth and evaluating the roles of various components of the adaptive immune response on viral pathogenesis and clearance in the 4–6-week-old B6 mouse model are warranted.

Although our study demonstrated that multiple clinical strains of CHIKV are capable of infecting the brain and spinal cord in mice, there was high variability in response, and clinical disease was not as severe as that seen with the control prototypic alphavirus, SINV TE. SINV TE is a recombinant SINV strain containing neuroadapted viral sequences [85], and neuroadaptation through serial virus passage in mice has been performed for alphaviruses in previous studies to enhance neurovirulence [86,87,88,89]. Additionally, previous work with the Ross strain of CHIKV, which has been passaged over 150 times in neonatal mice, resulted in successful CNS infection and pathology following IN inoculation in 5-week-old B6 mice [16]. Therefore, performing serial passages of CHIKV SL15649 to produce a neurologic mouse-adapted CHIKV strain in mice may produce a model that develops more severe and consistent neurological signs, and possibly allows for neuroinvasion following peripheral inoculation.

One of the major limitations of the B6 model is that we were only able to achieve CNS infection via direct IC inoculation. Alphaviruses are transmitted by mosquitos, so a peripheral infection route, such as SQ injection of the footpad, presents a more translatable approach for examining CHIKV neuroinvasion and neurological infection in humans. Viral mutations, such as in the E2 glycoprotein of SINV or SFV, have been shown to affect neuroinvasive potential during alphavirus infection [90,91], so it is possible that other CHIKV strains may be capable of neuroinvasion in adult B6 mice. Indeed, one group using adult C57BL/6J mice reported successful infection of the brain following SQ footpad inoculation with an alternative ECSA-lineage CHIKV strain isolated during a 2010 outbreak in New Delhi, with morbidity scores correlating with CHIKV RNA load in the brain in an age-dependent manner [17,18]. However, as the modification of blood brain barrier permeability [92,93,94] and alteration of the innate immune response and complement pathway [60,95] have allowed for enhanced alphavirus neuroinvasion, host factors almost certainly also play a role. Therefore, further evaluation of the potential for CHIKV to infect the CNS following peripheral infection using outbred mouse stocks, such as CD-1 mice, other inbred mouse strains, or systems genetics populations such as the Collaborative Cross or Diversity Outbred is warranted. Regardless, this study shows that 4–6-week-old immunocompetent CD-1 and B6 mice are capable of neurologic infection by direct inoculation with CHIKV, providing two mouse models by which to study CHIKV pathogenesis and the host immune response once the virus has successfully reached the CNS.

## Data Availability

All data applicable to this study are presented in the article and Supplementary File.

## Supplementary Materials

The following supporting information can be downloaded at: https://www.mdpi.com/article/10.3390/v15051057/s1, Table S1: qPCR Primer Probes.
