# Supplementary material for: Effect of Viral Strain and Host Age on Clinical Disease and Viral Replication in Immunocompetent Mouse Models of Chikungunya Encephalomyelitis
Source: Viruses. 2023 Apr 26;15(5):1057. doi: 10.3390/v15051057 (PMC10220978; doi:10.3390/v15051057)
Supplement: Supplementary file 1 [file viruses-15-01057-s001.zip › viruses-2322997-supplementary.pdf]

viruses

MDPI

Supplementary Material

Supplementary Table S1. qPCR Primer Probes.

Gene target	Company	Assay ID/Cat. #	Ref Seq
Gapdh	Applied Biosystems	4352932E	NM_008084(2)
Ifna4	IDT	Mm.PT.587678281.g	NM_010504(1)
Ifnb1	IDT	Mm.PT.58.30132453.g	NM_010510
Ifng	IDT	Mm.PT.58.41769240	NM_008337(1)
Il4	IDT	Mm.PT.58.7882098	NM_021283(1)
Il6	IDT	Mm.PT.58.10005566	NM_031168(1)
Il10	IDT	Mm.PT.58.13531087	NM_010548(1)
Il17a	IDT	Mm.PT.58.6531092	NM_0105532(1)
Tnf	IDT	Mm.PT.58.12575861	NM_013693(1)
